# Hypercapnia Alters Alveolar Epithelial Repair by a pH-Dependent and Adenylate Cyclase-Mediated Mechanism

**DOI:** 10.1038/s41598-018-36951-7

**Published:** 2019-01-23

**Authors:** Gustavo A. Cortes-Puentes, Blair Westerly, Dante Schiavo, Shaohua Wang, Randolph Stroetz, Bruce Walters, Rolf D. Hubmayr, Richard A. Oeckler

**Affiliations:** Department of Pulmonary and Critical Care Medicine, Mayo Clinic, Rochester, MN USA

## Abstract

Lung cell injury and repair is a hallmark of the acute respiratory distress syndrome (ARDS). Lung protective mechanical ventilation strategies in these patients may lead to hypercapnia (HC). Although HC has been explored in the clinical context of ARDS, its effect upon alveolar epithelial cell (AEC) wounding and repair remains poorly understood. We have previously reported that HC alters the likelihood of AEC repair by a pH-sensitive but otherwise unknown mechanism. Adenylate cyclase (AC) is an attractive candidate as a putative AEC CO_2_ sensor and effector as it is bicarbonate sensitive and controls key mediators of AEC repair. The effect of HC on AC activity and plasma membrane (PM) wound repair was measured in AEC type 1 exposed to normocapnia (NC, 40 Torr) or HC (80 Torr), ± tromethamine (THAM) or sodium bicarbonate (HCO3) ± AC probes in a micropuncture model of AEC injury relevant to ARDS. Intracellular pH and AC activity were measured and correlated with repair. HC decreased intracellular pH 0.56, cAMP by 37%, and absolute PM repair rate by 26%. Buffering or pharmacologic manipulation of AC reduced or reversed the effects of HC on AC activity (THAM 103%, HCO_3_ 113% of NC cAMP, ns; Forskolin 168%, p < 0.05) and PM repair (THAM 87%, HCO_3_ 108% of NC likelihood to repair, ns; Forskolin 160%, p < 0.01). These findings suggest AC to be a putative AEC CO_2_ sensor and modulator of AEC repair, and may have implications for future pharmacologic targeting of downstream messengers of the AC-cAMP axis in experimental models of ARDS.

## Introduction

The acute respiratory distress syndrome (ARDS) is a common cause of hypoxic respiratory failure that usually develops coincident with pneumonia, severe sepsis, aspiration, or trauma^1,2^. A subset of this entity, ventilator-induced lung injury (VILI), is recognized as a hospital-acquired condition and significant source of morbidity and mortality^2^ in patients exposed to mechanical ventilation at injurious settings. The hallmark of VILI is the mechanical disruption of the blood-gas barrier and widespread injury of resident alveolar cells that activate cellular stress, immune and inflammatory responses (collectively, “biotrauma”^3^). In turn, these mediators may drive the systemic sequelae of multiorgan system dysfunction and death. At their root, lung protective strategies (LPVS) are effective at least in part because they target a critical event in the pathogenesis of VILI – alveolar cell plasma membrane (PM) wounding by deforming stress^4^. Yet despite attempts to limit pathologic lung deformation by low tidal volume (V_T_)^5^ and increased positive-end expiratory pressure (PEEP)^6^ algorithms, incidence and mortality of these conditions remain unacceptably high.

The unintended consequences of LPVS include a reduction in minute ventilation and a worsening of ventilation-perfusion (VQ) mismatching that may lead to the development of hypercapnia (HC, defined as a PaCO_2_ > 45 mmHg) and hypercapnic acidosis (HCA, HC with a pH < 7.35). Historically HCA has been permitted as an adverse side effect of LPVS. However, the findings of several studies^7–13^ – including the ability of CO_2_ to alter susceptibility of the lung to wounding and repair independently of mechanically delivered tidal V_T_^9^ – challenge this convention and question whether “permissive” HC may in fact be “therapeutic” in ARDS. Conflicting evidence has only led to further controversy.

Previous investigations from our laboratory^14,15^ have suggested a deleterious, pH-mediated effect of HC on the repair of wounded alveolar epithelial cells (AEC). In those studies, unbuffered HCA inhibited^15^, while buffered HC restored^14^, repair. Yet the mechanism was not identified and the effects of CO_2_-mediated signaling on AEC repair pathways remain poorly understood. In the current study we explore the effect of pH on specific candidate signaling mediators of the HC regulation of AEC PM repair, and in so doing hope to identify potential therapeutic targets for future testing in experimental models of ARDS/VILI.

**Figure 1:**
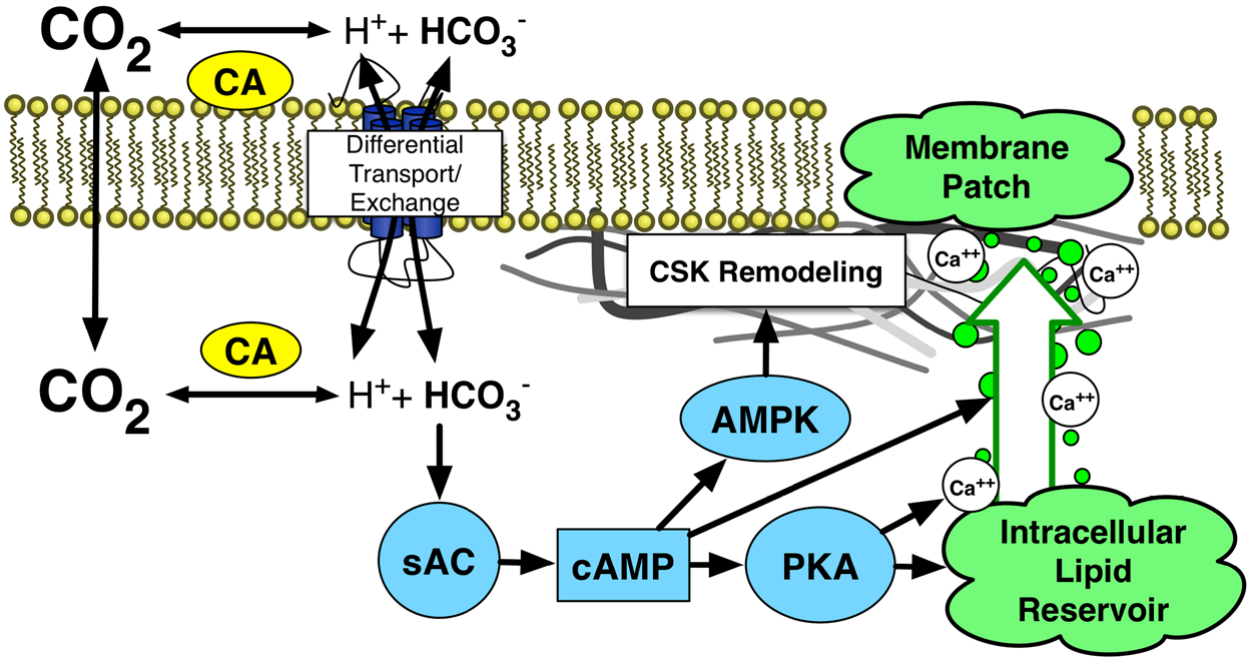
Hypothesized model for HC-mediated changes in AEC repair. CA, carbonic anhydrase; sAC, soluble adenylate cyclase; cAMP, cyclic AMP; PKA, protein kinase (**A**); AMPK, AMP-dependent protein kinase.

Adenylate cyclase (AC) is an attractive candidate to participate in the HC-mediated regulation of lung cell repair. AC has been proposed to be a CO_2_ and pH sensor in both the cytosolic and mitochondrial compartments of various mammalian cells^16^. The soluble isoform of AC has been demonstrated to be activated by the direct binding of bicarbonate (HCO_3_) to the enzyme, leading to increased cyclic AMP (cAMP) production^17,18^ and downstream second messenger activation related to repair (Fig. 1). In the cytosolic compartment cAMP may activate protein kinase A (PKA), exchange proteins activated by cAMP 1 and 2 (EPAC)^19^, and the cellular transcription factor cAMP response element-binding (CREB) which, through actions on GTP binding proteins (Rabs)^20^ and SNAP protein and SNARE complexing^21^, modulate endomembrane lipid vesicle production, trafficking, and PM docking^22^ integral to successful PM repair. Such processes are Ca^2+^ dependent, and PKA is well established to alter intracellular calcium through direct actions on Ca^2+^ channels and phosphoinositide-mediated release from intracellular stores. Cyclic AMP also activates adenosine monophosphate (AMP)-activated protein kinase (AMPK) and its downstream pathways to drive subcortical actin stress fiber formation and CSK rearrangement^23^. AMPK signaling has been implicated in osmotic-mediated CSK remodeling, which in turn has been demonstrated to decrease AEC PM wounding and death in an interfacial stress model of VILI^24^.

Here we propose AC to be both a putative CO_2_ sensor and master regulator of repair in AEC, and hypothesize that HC alters AEC PM repair through CO_2_-derived HCO_3_-dependent changes in AC-cAMP axis and downstream second messenger signaling (Fig. 1).

## Material and Methods

The use of rodents for cell isolation was previously approved by the *Mayo Clinic Institutional Animal Care and Use Committee (IACUC)*, as well as the *Mayo Clinic Institutional Review Board (IRB)*.

### Cell isolation and culture

Sprague-Dawley rat AEC type 1 (AT1) cells were isolated by immunoselection for T1-alpha by methods we have previously published^25^. Resultant AT1 were seeded on glass coverslips, cultured in high glucose Dulbecco’s Modified Eagle Media (DMEM) supplemented with 10% FBS, penicillin, streptomycin, and amphotericin (100 U/ml, 100 µg/ml, and 25 µg/ml, respectively; Sigma, St. Louis, MO), and maintained in a 5% CO_2_ humidified 37 C incubator until day of the experiment. All AT1 experiments were performed on day 5 post-harvest.

Human A549 cells (American Type Culture Collection, Manassas, VA) were cultured in Ham’s F-12K containing L-glutamine (2 mM), and FBS and penicillin-streptomycin-amphotericin B as described for AT1. A549 cells were passaged 48 h prior to each experiment, resulting in 85–90% confluent monolayers.

### Intracellular pH measurement

A549 were plated on a 96-well microplate to a concentration of 30,000 per well and incubated overnight. Cells were rinsed twice with Hanks Buffered Saline Solution (HBSS) and 2′7′-bis(2-carboxyethyl)-5-(and-6)-carboxyfluorescein (BCECF) acetoxymethyl (AM) was added at a concentration of 5 µM at 37 °C, under normocapnia (5% CO_2_, 40 Torr) for 30 minutes. Cells were then rinsed with HBSS and exposed to either normocapnia or hypercapnia (10% CO_2_ or 80 Torr), with or without increasing concentrations of bicarbonate or THAM, for 20 minutes. Cells were transferred to a microplate reader for fluorescence measurements. Excitation at 440 nm and 490 nm was performed and emission at 535 nm measured. The ratios of fluorescence intensity for each wavelength of excitation were calculated and inserted into a standard curve prepared as previously described^26^. Cells were rinsed and then exposed to a solution of 80 mM KCl in HBSS and nigericin titrated to a known pH with HCl or NaHCO_3_. Nigericin, a K+/H+ ionophore, equilibrates intra- and extracellular pH when the K+ concentration is equalized between intra- and extracellular compartments. The emission recorded at 535 nm after excitation at 440 nm and 490 nm resulted in a unique fluorescent ratios for a given (known) pH, and these values were then used to construct a standard pH reference curve^26^.

### Adenylate cyclase expression and activity

Day #5 AT1 maintained in complete DMEM were used for the studies. Cells were exposed to 20 minutes of NC or HC in the presence or absence of 1 mM HCO_3_, 10 mM THAM, or pretreatment with 100 mM ATP or 25 µM forskolin. AC activity was assessed by cAMP level measured by ELISA assay (Cayman Chemical, Ann Arbor, MI), and expressed as percent NC control.

### Micropuncture wounding model

A detailed description of this previously validated method for inducing focal PM injury can be found in Belete *et al*.^5^. Briefly, ATI between days 5 and 7 following isolation and plating onto glass-bottom dishes were loaded with 1 µM calcein AM for 1 h at 37 °C, washed, re-incubated with 1 mL of DMEM per coverslip, placed into a temperature and gas tension controlled chamber with real-time temperature and capnography monitoring, and mounted onto a motorized stage on an Axiovert S100 TV fluorescence microscope (Zeiss, Thornwood, NY). Pretreatment for 15 minutes with the desired CO_2_ level and/or experimental probe was performed prior to injury. The cell of interest was visualized with a 100x oil immersion lens, and a Hamamatsu Orca-ER CCD digital camera captured images at 1 fps. A motorized microinjector (InjectMan; Eppendorf, Hauppauge, NY) was used to position a 1 µm-diameter glass needle (Femtotip, Eppendorf) within the field of view above the targeted cell. The cell was then stabbed at a 45° angle with an injury duration of 0.3 s. PM integrity was inferred from the temporal decay in calcein AM fluorescence following micropuncture and was confirmed through the exclusion of propidium iodide (PI, 5 µg/ml) applied 3 min after injury. Rate of closure of successfully repaired AT1, i.e. The “reseal rate,” was calculated by post hoc analysis of the number of seconds until calcein fluorescence decay diminished below a previously validated threshold (<0.04%/s)^27^.

All methods were performed in accordance with the relevant guidelines and regulations.

## Statistics

Within condition effects were estimated using linear regression. Between condition effects were estimated by including the appropriate interaction term in a linear regression model adjusted for correlation of measurements within each experiment. In addition, we used Tukey correction for post hoc multiple comparisons. A p value < 0.05 was considered significant.

### Ethics approval and consent to participate

The use of rodents for cell isolation was previously approved by the Mayo Clinic Institutional Animal Care and Use Committee (IACUC), as well as the Mayo Clinic Institutional Review Board (IRB). Methods were carried out in accordance with the relevant guidelines and regulations.

## Results

### Intracellular pH measurements in AT1

**Figure 2:**
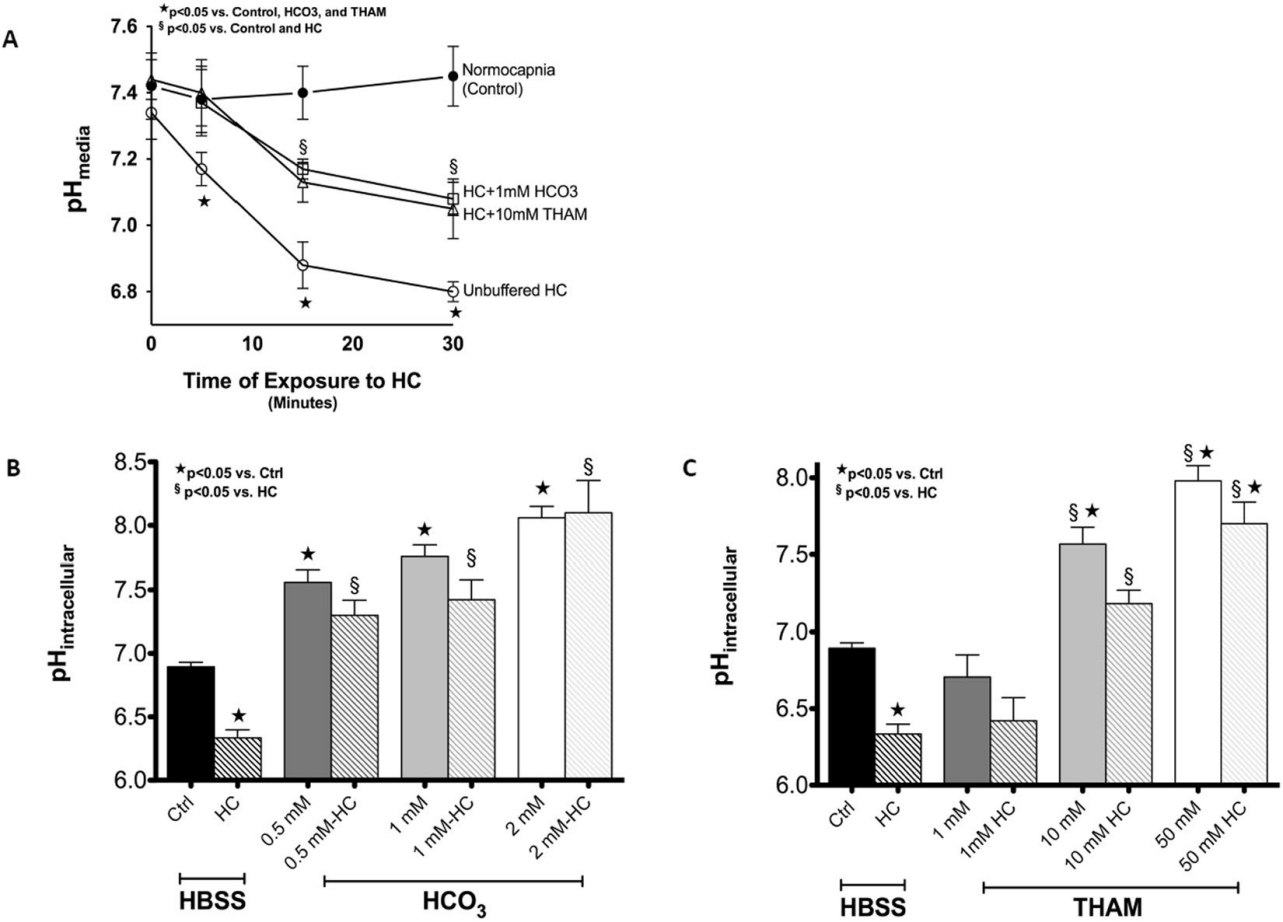
Milieu and intracellular pH measurements. Panel A: F12k serum-free media pH measured in the presence or absence of hypercapnia and buffering. Panels B and C: Intracellular pH as measured by BCECF technique (see Methods) in the presence or absence of hypercapnia, 1 mM NaHCO_3_ and 10 mM THAM buffering, respectively. Ctrl, 40 Torr normocapnic control; HC, 80 Torr CO_2_ hypercapnia, HCO_3_, sodium bicarbonate; THAM, tris-hydroxyaminomethamine.

Although literature demonstrating the acidification of serum pH *in vivo* in response to HC is fairly consistent, the effect of milieu pH – and the pharmacologic buffering thereof – upon intracellular pH (pH_i_) remains controversial and poorly characterized^28^. To establish the change in pH_i_ in AT1 due to exposure to HC, we first measured the pH of cell culture media DMEM and Hanks’s Buffered Saline Solution (HBSS) over time courses of exposure to 80 Torr (12%) HC (Fig. 2A). Due to the need to accurately measure and control buffer concentrations in further experiments, HBSS was preferred to DMEM due to the lack of premixed buffering agents (non-bicarbonate containing solution) while maintaining iso-osmotic (295–300 mOsm) characteristics. The pH of AT1 exposed to 40 Torr (5%) CO_2_ in HBSS for 15 minutes was 6.89 ± 0.011. Hypercapnia (80 Torr, 12%) decreased pH_i_ by a mean pH difference (MPD) of 0.5593 (95% CI 0.2108–0.9078, p < 0.05).

To determine the appropriate dose of either sodium bicarbonate (HCO_3_) or tromethamine (THAM) in future experiments, increasing concentrations of each agent were added to AT1 in HBSS under normocapnia (HCO_3_ MPD 0.6652, 95% CI 0.1856–1.145, p < 0.05 at 0.5 mM, 0.8617, 95% CI 0.4041–1.319, p < 0.05 at 1 mM, and 1.165, 95% CI 0.6856–1.645, p < 0.05 at 2 mM (Fig. 2B); THAM MPD 0.6741, 95% CI 0.290–1.058, p < 0.05 at 10 mM and 1.087, 95% CI 0.6841–1.489 p < 0.05 at 50 mM (Fig. 2C)) and hypercapnia (HCO_3_ MPD 0.9637, 95% CI 0.4551–1.472, p < 0.05 at 0.5 mM, 1.087, 95% CI 0.6073–1.566, p < 0.05 at 1 mM, and 1.764, 95% CI 1.255–2.273, p < 0.05 at 2 mM (Fig. 2B); THAM MPD 0.8500, 95% CI 0.4475–1.252, p < 0.05 at 10 mM and 1.366, 95% CI 0.9637–1.769, p < 0.05 at 50 mM (Fig. 2C)). After statistical analysis of 10 separate experiments, optimal dosing was determined to be a final concentration of either 1 mM HCO_3_ or 10 mM THAM.

### Cellular cAMP measurement

**Figure 3:**
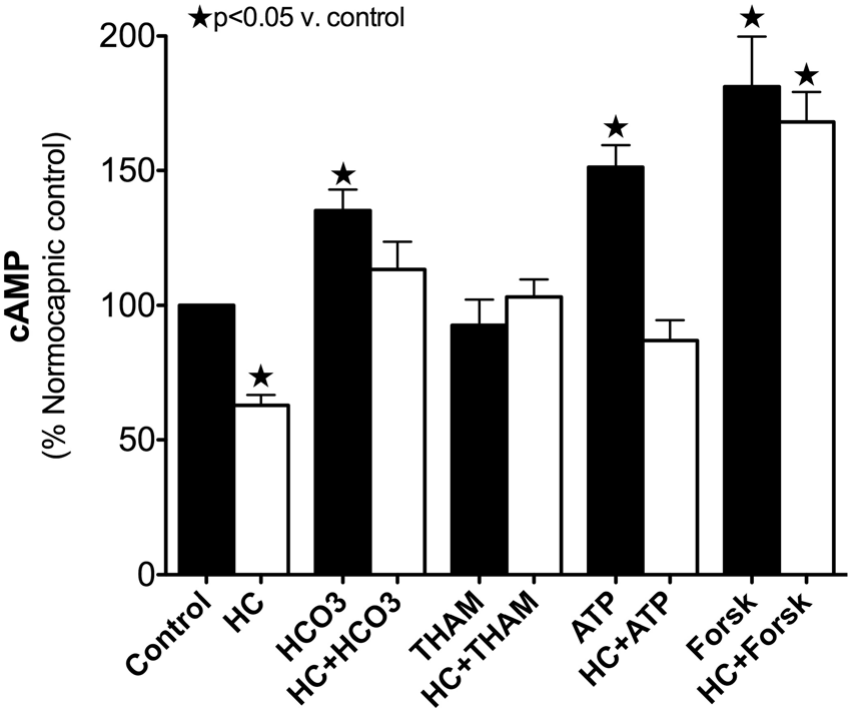
Effects of hypercapnia and buffering on adenylate cyclase activity. Cyclic AMP level in pM (see text) was measured after 15 minute exposure to normo- or hypercapnia in the presence or absence of 1 mM sodium bicarbonate (HCO_3_), 10 mM tris-hydroxyaminomethamine (THAM) buffering, 100 mM adenosine triphosphate (ATP), and/or the stimulation of adenylate cyclase signaling by the diterpene analogue forskolin (Forsk). The results are reported as percent of normocapnic control cAMP level.

Baseline NC cAMP level was 13.72 ± 2.04 pM (n = 4), and this decreased to 11.4 ± 1.8 pM after 5 minutes, and to 8.62 ± 0.7 pM (63% control) after 15 minutes of exposure to HC (Fig. 3). The HC-mediated inhibition in AC activity was prevented by the addition of HCO_3_ (15.50 ± 4.03 pM, 113% control) or THAM (14.13 ± 1.84 pM, 103% control) buffer just prior to the exposure to HC. The addition of HCO_3_ (18.52 ± 2.87 pM, 135% control, p < 0.05), but not THAM (12.74 ± 2.42 pM, 93% control, ns) to normocapnic A549 increased cAMP levels. Providing substrate for the adenylate cyclase enzyme (ATP) lead to increased cAMP production under NC (20.76 ± 3.3 pM, 151% control, p < 0.05) but not HC (11.94 ± 1.79 pM, 87% control). Stimulation of several isoforms of AC by the diterpene analog forskolin increased cAMP irrespective of CO_2_ (NC: 24.84 ± 9.1 pM, 181% control, p < 0.05; HC: 23.05 ± 5.3 pM, 168% control, p < 0.05).

### Micropuncture PM wound reseal analysis

**Figure 4:**
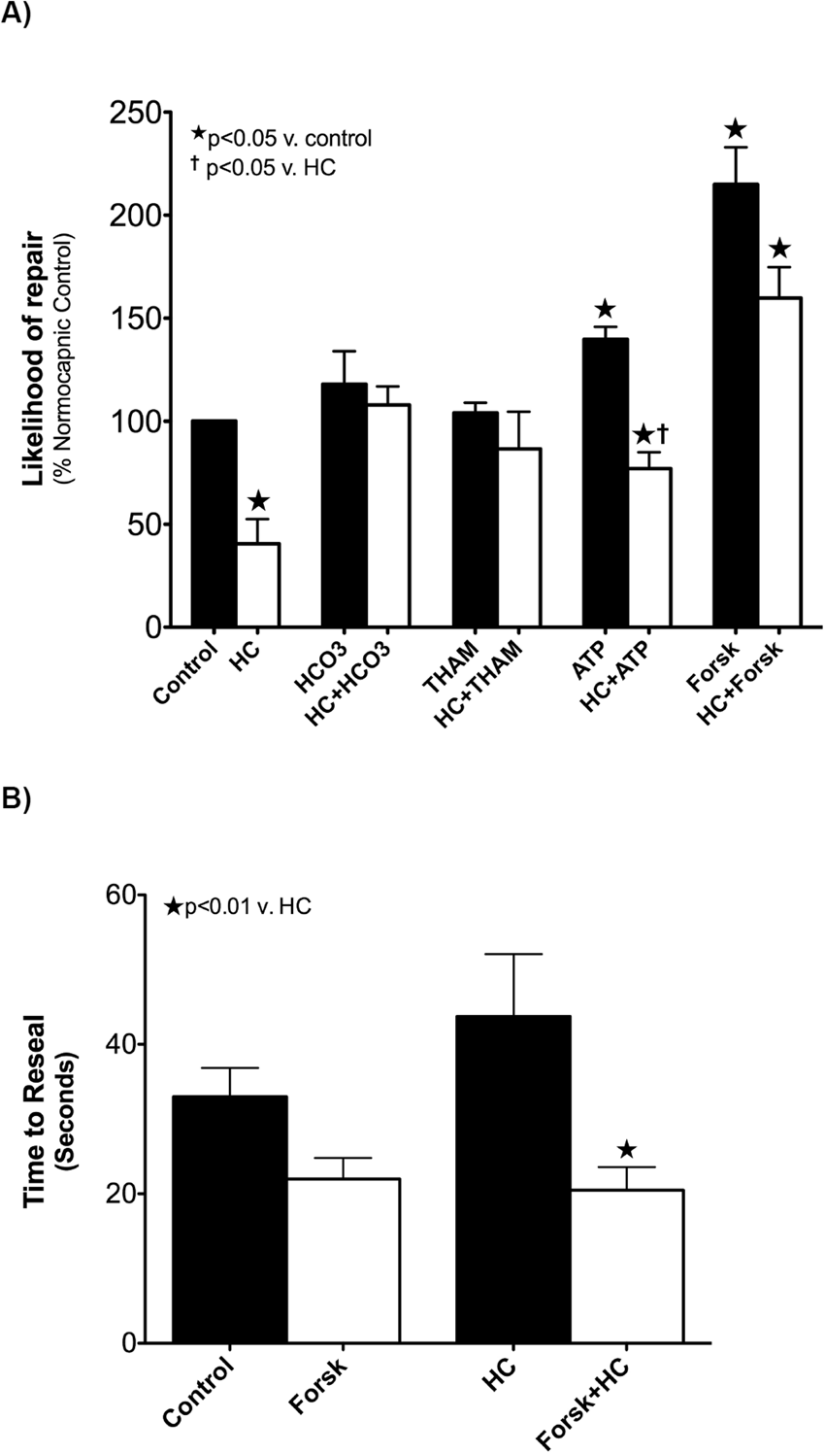
Measurement of AEC PM repair in the presence of hypercapnia, buffering, and AC manipulation. Panel A: Likelihood of PM repair and cell survival compared to normocapnic control in AT1 exposed to hypercapnia (HC), 1 mM sodium bicarbonate (HCO_3_), 10 mM tris-hydroxyaminomethamine (THAM) buffering, 100 mM adenosine triphosphate (ATP), and/or adenylate cyclase activation by the diterpene analogue 25 µM forskolin (Forsk). Panel B: PM reseal rate, in seconds, under NC or HC, and the presence or absence of adenylate cyclase activation by forskolin.

In preliminary experiments we found HC impaired PM wound reseal rates in both A549 (HC 58% vs. NC 72% wounded cells resealed) and AT1 (HC 57% vs. NC 77% wounded cells resealed). Further data shown are for subsequent AT1 cell experiments. To compare the rate of repair across groups, the percent of wounded and repaired cells were recorded and data for each experimental group expressed as the likelihood of repair compared to NC controls (Fig. 4A). Compared to NC control, the likelihood that AT1 exposed to HC would repair was decreased (40.6 ± 12%, p < 0.01) and buffering HC with either HCO_3_ 107.9 ± 9% or THAM 86.6 ± 2% reversed this effect. Stimulation of AC by forskolin improved the likelihood of PM reseal and cell viability no matter the CO_2_ (NC 215 ± 18% and HC 159.8 ± 15%, p < 0.01). Providing substrate for AC (ATP) led to increased likelihood of repair under NC, but not HC, conditions (139.7 ± 6%, P < 0.01, and 77 ± 8%, ns, respectively).

**Figure 5:**
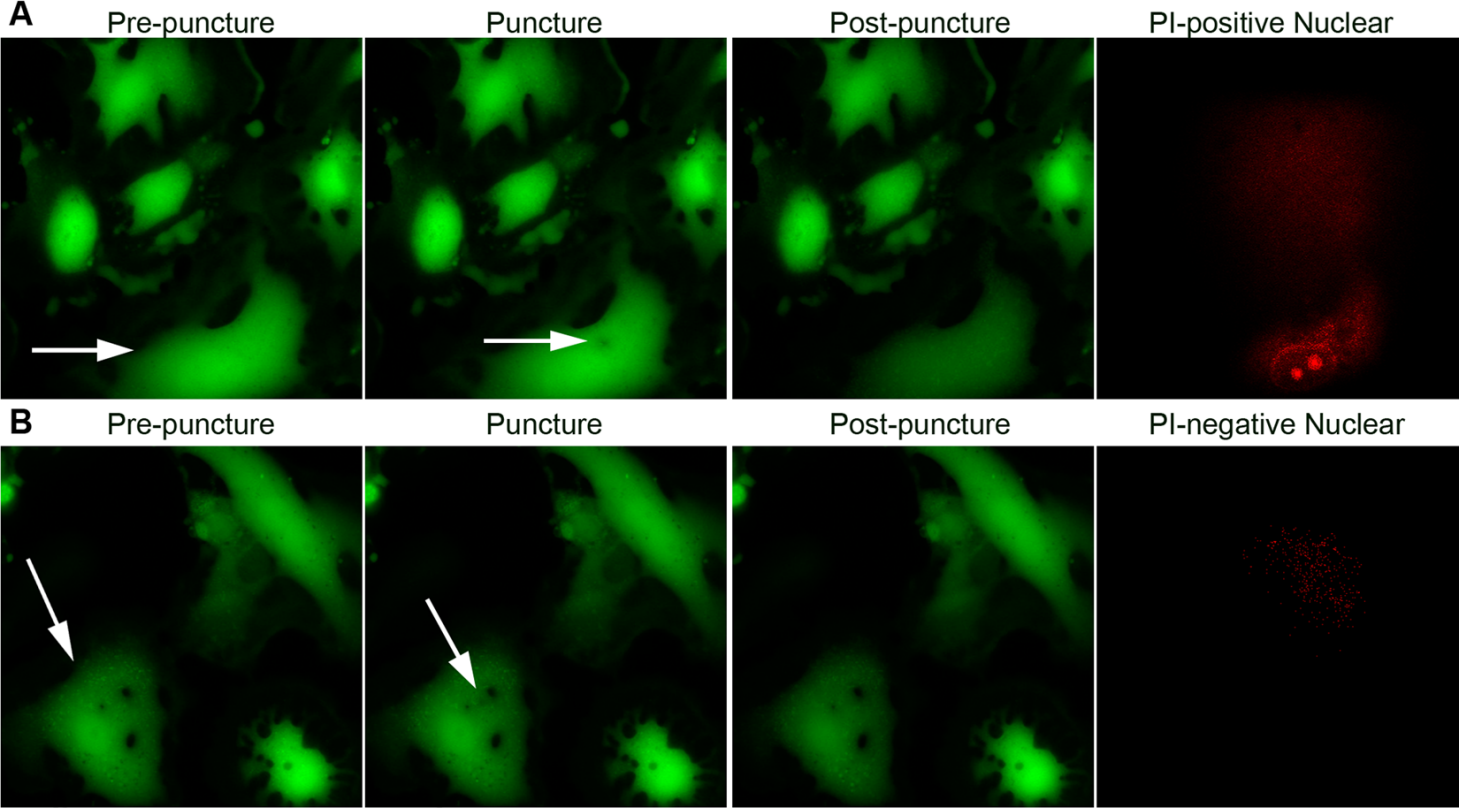
Representative images of AT1 cells injury and repair processes. Panel A shows serial images of an injured cell without repair: an AT1 cell (Arrow) at time of pre-puncture, puncture (arrow points the micropuncture wound), post-puncture and PI-positive cell nucleus after 3 minutes of repair time. PI-positive cell nucleus confirmed this cell was lethally wounded. Panel B shows serial images of an injured and repaired cell: an AT1 cell (arrow) at time of pre-puncture, puncture (arrow points the micropuncture wound), post-puncture and PI-negative cell nucleus after 3 minutes of repair time. PI-negative cell nucleus confirmed this cell was repaired after injury. Propidium iodide (PI, 5 µg/ml).

It has been shown that a subsequent wound to a previously injured cell repairs faster^29^, and that this potentiation appears to be mediated by downstream effectors of cAMP including CREB^30^. Although AEC rarely survive if reseal time is prolonged significantly ( > 60–120 s), it remains unknown whether ultimate cell viability after wounding is related to the speed at which PM wounds reseal. Post-hoc analysis of time from micropuncture wound to PM reseal was found to be consistent with previously reported reseal rates under normocapnic conditions, with average time to PM wound closure 33 ± 7.7 s (Fig. 4B). There was a trend toward HC retarding the reseal rate (43 ± 16.7 s, p = 0.07), that did not reach significance. However, the pharmacological activation of adenylate cyclase by forskolin consistently reduced reseal times under both NC (22 ± 5.6 s, p < 0.01) and HC (20.5 ± 6.1, p < 0.01). Representative images of AT1 cell injury and repair process are shown in Fig. 5.

## Discussion

In the current study we find AC to be a putative CO_2_ sensor in AEC, and demonstrate that the physicochemical transduction of CO_2_ gas tension into a cAMP second message by AC ultimately modulates PM wound repair. By live cell imaging we show that exposure to a physiologically relevant level of hypercapnia for as little as 15 minutes results in milieu and intracellular acidosis, a reduction in intracellular cAMP level, and a decreased likelihood of injured cell viability. Through buffering, but not the addition of ATP, AC activity at any given CO_2_ level is facilitated, suggesting that the AC isoform(s) involved in the response to HC may be either recruited or further stimulated by a relative excess of HCO_3_. These data are consistent with previous findings in the neuronal literature of a direct activation of specific AC isoforms by bicarbonate^17,18,31^, and emphasize the complex, inter-related nature of CO_2_-HCO_3_-pH as defined by Henderson-Hasselbach.

### Effect of HC on pH_i_ in AT1

Although our reported findings with regard to HC’s effect on pH seem intuitive, previous studies have reported divergent pH_i_ responses to milieu acidosis. In one review, a majority of twenty studies in cellular, small and large animal models of ARDS as well as clinical ARDS patients demonstrated either no change or a paradoxical increase in pH_i_ in response to serum acidosis^28^. This was attributed to the acute dissociative effects of NaHCO_3_ and – relevant to ARDS – the effect of ventilation limitation on buffer efficacy. Mechanistically, NaHCO_3_ may transiently increase intracellular CO_2_ due to carbonic anhydrase (CA) activity in the PM and milieu (Formula 1), while THAM is a strict proton acceptor with no such effect. Although not explored in the current study, the role of CA in the rapid transport of CO_2_, H^+^, and HCO_3_ between intra- and extracellular compartments^32,33^ and differential transport of H+ and HCO_3_ by symporter (e.g. Na/HCO_3_) and antiporter (e.g. Na/H) mechanisms further complicate matters.

1 $$C{O}_{2}+{H}_{2}O\mathop{\leftrightarrow }\limits^{\,CA\,}{H}_{2}C{O}_{3}\mathop{\leftrightarrow }\limits^{\,CA\,}{H}^{+}+HC{O}_{3}$$

The chronicity of exposure adds yet an additional layer of complexity. It is likely that non-steady state responses assessed in our acute model of HC may be quite different from those of steady state conditions, and chronic exposure may change the expression of key signaling effectors. In the case of the AC-cAMP axis, we have measured an increase in soluble AC protein and mRNA expression after 6 and 12 hours of exposure to HC in A549 (Oeckler, unpublished data).

The rapid change in pH_i_ induced by HC, HCO_3_, or THAM highlights a potential pitfall in trying to reconcile the data regarding the “permissive” or “therapeutic” nature of HC in ARDS. Has HCO_3_ been overlooked as a signaling molecule? Might the choice of media or fluid selection influence experimental outcome? The composition of commercially available media varies significantly with regard to buffer capacity. For example, DMEM contains on average 3.7 g/L (44 mM) NaHCO_3_, while F12k only 2.5 g/L (30 mM) NaHCO_3_, without taking into consideration the additional buffer capacities of ‘complete’ (serum protein-added) media, non-sodium salts, chelators, and proton acceptors present in or added to experimental solutions. Taking in to account such differences in buffer capacity, it is conceivable that a similar exposure to a similar level of HC in one experimental model utilizing complete DMEM and another F12k might result in significantly different local AEC pH_i_ and activity of pH-sensitive, calcium-dependent signaling mechanisms including those that regulate the repair processes described here. These findings have implications for ventilatory and fluid management strategies in future experimental models of ARDS.

### AC as HC sensor and effector

The inhibition of the AC-cAMP axis by HC is restored by buffering, consistent with a role for the AC axis as both a pH-mediated CO_2_ sensor, and “master” effector through broad-reaching downstream second messenger cascades known to ultimately alter regulation of calcium and calcium release necessary for trafficking processes. The ability of both HCO_3_ and THAM to increase cAMP suggests that either an absolute (by HCO_3_ administration) or relative (by THAM sequestration of H^+^) excess of HCO_3_, rather than CO_2_ *per se*, drives the change in AC-cAMP signaling. In other words HCO3 level acts as a throttle; That HCO_3_ is known to directly bind and activate soluble AC (sAC), makes this ubiquitous isoform an attractive candidate as the primary source of cAMP in our model.

### The AC-cAMP axis and PM wound repair

Consistent with previously published reports, we find that HC inhibits the repair of wounded AEC, and that -as described above- this appears to be due to inhibition of a CO2-AC-cAMP dependent signaling pathway that is constitutively active and substrate limited. Acute hypercapnia, due to a relative paucity of HCO3, throttles AC production of cAMP signaling and retards PM resealing and repair. Although speculative, states resulting in hypercapnia are also likely to lead to reduced cellular energy stores and ATP availability, further limiting substrate, and throttling this axis.

ATP has well-established roles in other AEC repair pathways through auto- and paracrine purinergic signaling. The combination of providing more substrate and increased purinergic (P2Y2) signaling may explain the findings with ATP under both NC and HC. Under NC, substrate limitation appears to be the primary driver of AC activity. In HC, where AC activity is inhibited and unable to be overcome without buffering, the activation of complimentary purinergic-dependent repair pathways by the direct administration of ATP may partially – but not fully restore – PM repair in the absence of necessary trafficking and other cAMP-dependent mediators of repair. The discordant effects of ATP supplementation under NC and HC provide further evidence that AC is constitutively, yet sub-maximally active and substrate limited under baseline conditions. Alternatively, it could be that the doses of HCO_3_ (1 mM) and THAM (10 mM) used, although equivalent in ability to buffer media pH under HC (Fig. 2A), had unequal effects on pH_i_ (MPD 0.86 HCO_3_ v. 0.67 THAM under NC, and 1.09 v. 0.85 MPD under HC, Fig. 2B,C, respectively). At all doses pH_i_ was slightly more alkaline (higher HCO_3_:H+) and AC activity was higher in the NaHCO_3_ group.

Finally, the activation of the AC-cAMP axis by forskolin increased the speed and likelihood with which repair took place. Most cells reseal PM wounds within 30–40 seconds^27,29^, and we have previously observed that PM wounds not repaired within 90–120 seconds are uniformly fatal. Yet it remains unclear whether or not a reduction in time-to-reseal of the average cell will increase its likelihood of survival. Although causality cannot be inferred from our limited data on time-to-reseal, there was a very strong correlation between cAMP, faster reseal rate, and likelihood of PM wound repair in AT1 under all conditions. Whether this is related to speed, the activation of complementary repair pathways, both, or a yet unidentified pathway remains unclear. Nonetheless, these findings demonstrate that the AC-cAMP signaling axis is integral to AEC repair, and further dissection of downstream signaling elements may provide targets for pharmacologic manipulation of repair to be explored in future experimental models of ARDS.

### Limitations

The physical chemistry of buffer composition and interaction at the air-liquid and PM-milieu interfaces is complex, and further complicated *in vitro* by varying buffer concentrations and chemistries in ‘off the shelf’ cell culture media, as well as whether or not serum protein (“complete media”) was used and at what dose. Because of this, we utilized commercially available balanced salt solutions without pre-mixed buffer such that the exact dosing of HCO_3_ or THAM added for each experiment lead to a known final buffer concentration at normal osmolarity (290–300 mOsm). Differentiation of soluble, membrane-bound, and compartmentalized AC isoforms 1–10 is hindered by the lack of specific pharmacologic probes. Although sAC is the only isoform confirmed in the literature to be HCO_3_ sensitive, the improvement in repair seen by forskolin, an activator of membrane bound but not soluble AC, suggests that both sAC and one or more of the other isoforms may play a role. Significant controversy exists regarding the specificity and efficacy of a number of adenylate cyclase inhibitors in the literature, with a recent study demonstrating most non-specific or completely ineffective^34^.

### Future direction

The observations in the current study are exciting for two reasons. First, CO_2_ is ubiquitous, easily measured, and readily manipulated through therapeutic interventions in the clinical setting. The ability to exploit HC to modulate cellular repair by “turning the ventilator knobs” or altering intravenous fluid supplementation or buffer could have far-reaching and relevant clinical consequences.

Second, these observations inform future studies in cell and small animal models of ARDS and VILI. Soluble AC exists in the mitochondrial compartment and can directly enhance cellular respiration through stimulation of the catalytic activity of complex I^35^, EPAC-2 mediated ATP production (reviewed in^19^), and through PKA- and CREB-mediated alterations of complex I expression^36^. Resultant ATP may exert autocrine and paracrine effects through purinergic P2Y2-receptor signaling, and this in turn has recently been implicated in the potentiation of lysosomal exocytosis (lipid trafficking) necessary for PM wound repair^37^. The extensive second messaging influence of AC through cAMP second-messengers PKA, AMPK, and EPAC-1 in the cytosolic compartment ought to be investigated due to their roles in lipid trafficking, vesicle docking, and cytoskeletal rearrangement important for successful PM reseal and repair.

### Conclusion

The current study demonstrates that the AC-cAMP axis in lung epithelia is CO_2_-sensitive and pH-dependent, implying a role for AC as a putative AEC CO_2_ sensor with relevance to multiple cAMP-dependent cellular processes. HC inhibits, while buffering of HC restores, the AC-cAMP axis and the likelihood of injured cells to repair. The modulation of wounded AEC repair by HC and buffering highlights the potential influence of ventilatory and fluid management choices on injury models of ARDS. The ability to dissect and pharmacologically manipulate the downstream mediators of the AC-cAMP axis may provide for a more precise, targeted approach to augment repair in future experimental models of VILI.

## Data Availability

Authors declare no restrictions on the availability of materials or information at the time of submission.
